# The impact of structure adjustment of energy consumption on the structural upgrading of the construction industry

**DOI:** 10.1371/journal.pone.0334359

**Published:** 2025-10-31

**Authors:** Zenan Qin, Junwei Li

**Affiliations:** 1 School of Finance, Harbin University of Commerce, Harbin, China; 2 Business School, Sichuan University, Chengdu, China; 3 School of Accounting and Finance, Hong Kong Polytechnic University, Hung Hom, Hong Kong; Policy Resaerch Institute, Government of Nepal, NEPAL

## Abstract

Using spatial Durbin model on panel data from 30 provinces in China from 2008 to 2022, this article explores the technical effect, price effect, and spatial effect of energy consumption structure on the structure of the construction industry. It is found that structure adjustment of energy consumption (SAEC) has a significant promoting effect on structural upgrading of the construction industry (SUC), exhibiting a negative spillover effect in space. Technology spillovers and research and development (R&D) are also the main channels through which SAEC affects SUC, while the channel effect of energy price effect is not significant. Further research has found that reducing the proportion of coal, oil, and natural gas consumption can promote SUC, with natural gas having the strongest effect. At the regional level, SAEC has a stronger promoting effect on SUC in the central and western regions, but its spatial negative spillover effect is more pronounced in the eastern region; On the temporal level, the promotion effect of SAEC on SUC was stronger from 2008 to 2014, while its spatial negative spillover effect was more pronounced from 2015 to 2022. In addition, with the increase in energy prices and human capital levels, it can further stimulate the promoting effect of SAEC on SUC.

## 1. Introduction

China’s economy has transitioned from high-speed to high-quality growth, making structural upgrading imperative [1,2]. The construction industry, which contributes 7% of GDP and absorbs nearly 30% of national energy use, is simultaneously tasked with alleviating employment pressure and achieving the “dual-carbon” goal [3,4]. Yet the prevailing energy mix—dominated by coal, oil and natural gas—remains carbon-intensive and is widely believed to constrain the sector’s shift toward green, specialized and high-tech production [5,6]. Whether, how and to what extent the structure adjustment of energy consumption (SAEC) drives the structural upgrading of the construction industry (SUC) therefore constitutes a critical but unresolved question.

Empirical studies have relied on three main analytical tools: (1) input–output or optimization models to quantify energy-saving potential from industrial restructuring [7,8]. (2) index-decomposition techniques such as LMDI to trace energy-structure effects [9,10]. (3) provincial or national panel data sets drawn from China Energy Statistical Yearbook, China Industrial Economy Statistical Yearbook and China Construction Industry Statistical Yearbook to estimate elasticity or causality between energy variables and industrial performance [11,12]. These works, however, rarely integrate spatial dependence or sector-specific upgrading metrics.

Early international evidence indicates that foreign entry can enhance host-country construction market structure [13]. Tian et al. (2017) empirically verified that industrial structure adjustment reduces energy consumption by 19% through factor reallocation, supported by panel data from 30 Chinese provinces [14]. Regarding energy-to-industry causality, Woo (2018) finds that deteriorating energy conditions hinder industrial upgrading in Korea [15], whereas Sajadifar and Mohamadbagheri (2018) establish a long-run nexus between aggregate energy use and industrial structure in fifteen developing economies [16]. Zhu and Shan (2020) further reveal an inverted-U relationship between sectoral energy intensity and output share for heavy, light and knowledge-intensive industries [17]. Collectively, these studies confirm that energy and industry co-evolve, but they stop short of unpacking the mechanisms inside the construction sector.

The European Union has mandated through the Energy Performance of Buildings Directive that new buildings adopt renewable energy sources (such as solar energy and geothermal energy) and established a “green building certification system” to promote the transformation of the construction industry from reliance on high-carbon energy to low-carbon energy [18]. The United States provides tax incentives for new energy building projects through the Energy Policy Act, while encouraging cooperation between construction enterprises and energy companies in research and development of low-carbon technologies. Its experience shows that the combination of “policy mandatory measures+market incentives” can effectively accelerate structural upgrading [19]. India has included the new energy utilization rate in the construction industry into the assessment indicators of local governments through the “National Solar Energy Plan” to force high-energy-consuming construction enterprises to transform. However, due to insufficient technical reserves, a “policy implementation gap” has emerged [20]. Brazil, on the other hand, has replaced traditional fossil energy with biomass energy (such as electricity generated from bagasse), reducing the construction industry’s dependence on imported energy. Its model of “local energy+construction industry linkage” has reference value for central and western regions of China [21]. International experience shows that policy effects are closely related to the energy endowments of various countries (such as resource reserves and technical level). China needs to take into account its energy structure characterized by “abundant coal, poor oil, and little gas”, avoid blindly copying the high-subsidy models of Europe and the United States, and instead focus on the combination of differentiated regional policies and independent technological innovation [22].

Government interventions play a critical role in mediating the relationship between energy structure adjustment and industrial upgrading. Existing studies have highlighted that policy tools such as energy quotas, carbon pricing, and green subsidies can accelerate fossil energy substitution [23,24]. For the construction sector specifically, Franco et al. (2021) found that government-mandated green building standards significantly promote the adoption of low-carbon technologies [25], while Lin and Zhou (2021) emphasized that regional industrial policies can mitigate spatial imbalances in structural upgrading [26]. However, few studies have systematically analyzed how these interventions modulate the technical, price, and spatial effects of SAEC on SUC, leaving a critical theoretical gap.

Despite these advancements, four critical gaps remain: First, the construction industry, despite its energy intensity, has received scant attention in the energy-structure literature. Second, existing analyses largely ignore spatial spillovers arising from cross-regional factor mobility and asymmetric regulation, precluding a full picture of SAEC impacts. Third, measurement of industrial upgrading remains coarse; few papers adopt firm-level specialization ratios to proxy SUC. Lastly, endogeneity between energy structure and industrial upgrading is seldom addressed, leaving causal inference open to challenge.

This paper therefore aims to (1) theorize and empirically test the technical, price and spatial channels through which SAEC affects SUC; (2) apply spatial econometric models to 30-province panel data while controlling for endogeneity via instrumental variables; and (3) examine heterogeneous effects by energy type, region and time period. The findings are expected to inform targeted policies, such as differentiated energy quotas, regional coordination schemes and human-capital initiatives, that simultaneously accelerate the green transformation of energy use and the high-quality upgrading of China’s construction industry.

This paper contributes to the journal’s focus on ‘energy transition and industrial high-quality development’ by unpacking the mechanisms through which energy structure adjustment drives the construction industry’s upgrading, with specific attention to policy interventions and spatial coordination—key issues for sustainable development in emerging economies.

## 2. Theoretical mechanism

Energy is both a consumer good and an input factor, running through every aspect of economic operation. Based on the relevant literature mentioned above, this article analyzes the mechanism of SAEC on SUC from three aspects: technical effect, price effect, and spatial effect.

### 2.1 Technical effect

The technical effect of SAEC on SUC depends on the relative size of technical progress effect and technical rebound effect.

(1) The technical progress effect. The SAEC will make it difficult for construction enterprises to meet their fossil energy needs, and they will develop, introduce, and use advanced technologies to reduce energy consumption per unit output [27]. As consumers’ income levels continue to rise, their requirements for the quality of the construction industry will also increase. In order to meet consumers’ demand for construction products, construction enterprises will continuously improve their technical level and further promote the technical progress of the entire construction industry.
(2) The technical rebound effect. While SAEC drives technical progress (e.g., energy-efficient construction equipment or low-carbon building materials), it may inadvertently weaken incentives for long-term structural upgrading through a rebound mechanism. For example, if construction enterprises adopt energy-saving technologies that reduce fossil energy costs per unit output, they might expand production scales (e.g., increasing the number of high-energy-consuming projects like traditional concrete production) instead of shifting to low-carbon sectors (e.g., prefabricated building systems). This expansion reinforces reliance on existing fossil energy-dependent production models, creating path dependence and offsetting the intended upgrading effect of SAEC. Thus, technical progress induced by SAEC can have countervailing impacts: promoting efficiency in the short term but hindering structural transformation if the rebound effect dominates.

### 2.2 Price effect

Price effect refers to the total impact of changes in fossil energy prices on the energy purchases of enterprises (fossil energy, new energy), including income effect and substitution effect.

(1) The income effect. The strengthening of SAEC has limited the fossil resources used in the construction industry for production, resulting in a decrease in the supply of production factors, which will lead to an increase in production costs and a decrease in the actual income level of enterprises. Some construction enterprises will reduce their investment scale or exit the market [28]. It will induce the transfer of factors such as technology, labor, and capital, which is conducive to promoting the SUC.
(2) The substitution effect. The changes in the relative prices of fossil fuels and new energy will encourage enterprises to replace fossil fuels with new energy, increase their demand for new energy, and drive the development of the new energy industry. It includes links such as raw material supply, component production, complete machine assembly and manufacturing, and energy utilization and conversion. Among these links, the optimal products that should be developed in the construction industry are derived, such as emerging construction industries and high-tech industries, promoting the SUC.

### 2.3 Spatial effect

The spatial effect refers to the impact of SAEC on the structural changes of the construction industry in adjacent areas, including positive spillover effect and negative spillover effect.

(1) The positive spillover effect. Energy consumption assessment is a binding indicator for the development of the national economy. If a certain area strengthens the SAEC, it is highly susceptible to being imitated by neighboring areas. Adjacent regions have obvious advantages in experience learning, factor flow, and industrial coordinated development, which can to some extent weaken the negative impact of SAEC on the construction industry structure and promote the coordinated improvement of the construction industry structure between regions.
(2) The negative spillover effect. In reality, the SAEC often exhibits asymmetry among regions, while reducing the proportion of fossil energy consumption increases the production costs of the high-energy consuming construction industry, which naturally shifts towards neighboring regions with relatively loose energy consumption regulations. In contrast, the high-tech construction industry is less affected, making it easier to gain comparative advantages in green development and attract a large amount of production factors (such as material capital and human capital) to flow into the industry [29]. Driven by the cyclic cumulative causal relationship, high-energy consuming construction enterprises and high-tech construction enterprises will flow in opposite directions, leading to significant differentiation in the structure of the construction industry between regions.

**Fig 1:**
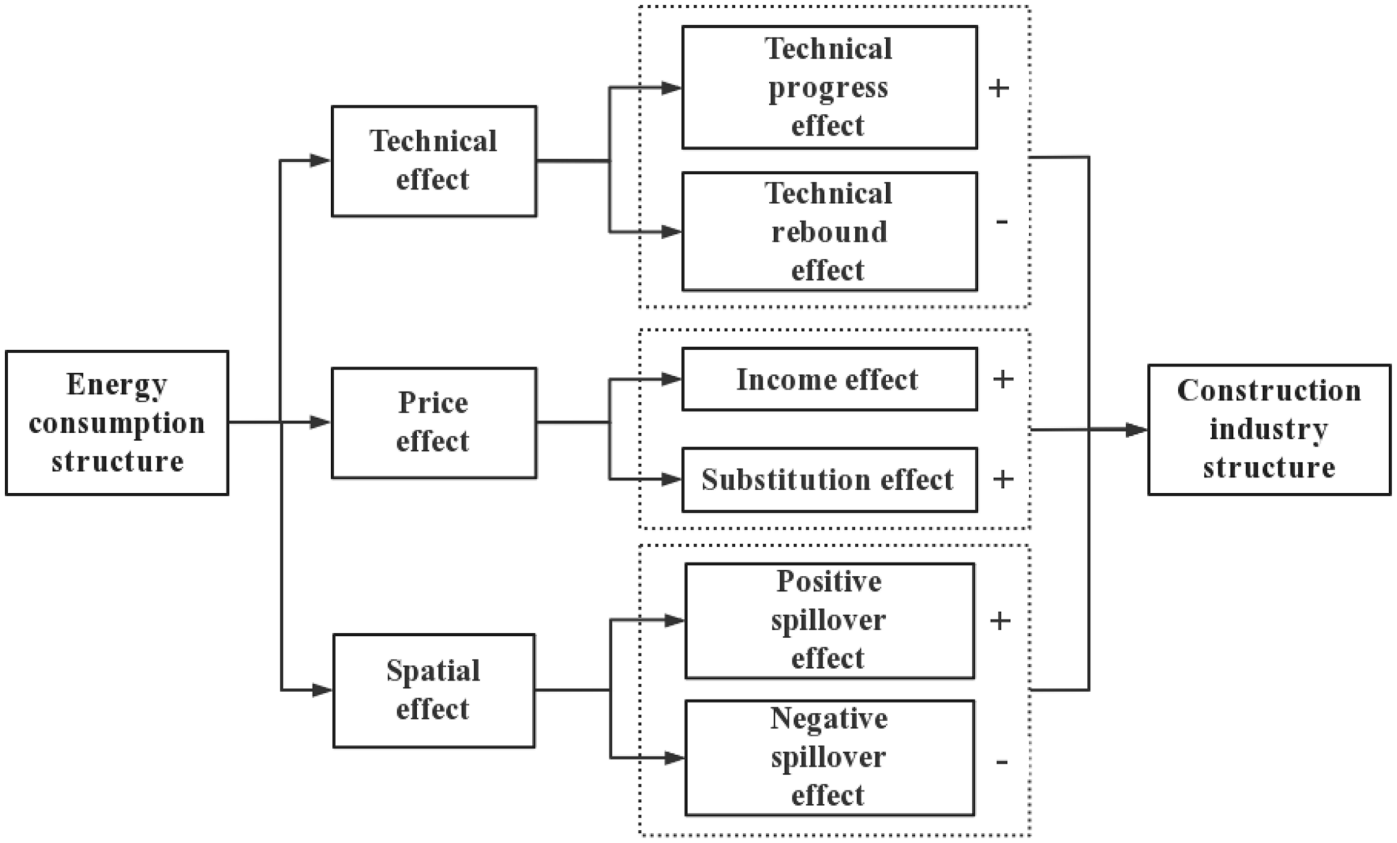
Theoretical mechanism of the impact of SAEC on SUC.

Fig 1 illustrates the three channels through which SAEC affects SUC. Firstly, technical effect. Driven by SAEC, construction enterprises develop energy-saving technologies (technical progress effect), but may also expand high-energy activities due to reduced costs (technical rebound effect). Secondly, price effect. Changes in energy prices (e.g., fossil fuel price hikes) trigger income effects (shrinking profits for energy-intensive firms) and substitution effects (shifting to new energy and high-tech sectors). Thirdly, spatial effect. SAEC in one region may induce positive spillovers (neighboring regions imitating green practices) or negative spillovers (high-energy firms relocating to regions with lax regulations).

## 3. Model setting and indicator selection

### 3.1 Model setting

For empirical analysis, this article establishes the benchmark model, as shown in Eq.(1):


$$SUC_{i,t}=c+\beta SAEC_{i,t}+\sum_{n=1}^{k}\alpha_{n}X_{n,i,t}+\delta_{i}+\mu_{i}+\varepsilon_{i,t}$$ (1)

Where i represents the region, t represents the year, c is a constant term, β is corresponding regression coefficient of the SAEC, k is the number of control variables, $X_{n,i,t}$ represents the n-th control variable in year t of the i-th region, $\alpha_{n}$ is the regression coefficient of the corresponding control variable, $\delta_{i},\mu_{i},\varepsilon_{i,t}$ represent individual fixed effects, time fixed effects, and random error terms, respectively.

Based on Eq.(1), set the following spatial econometric model, as shown in Eq.(2):


$$\begin{array}{c} SUC_{i,t}=c+\beta SAEC_{i,t}+\sum_{n=1}^{k}\alpha_{n}X_{n,i,t}+\theta \sum_{j=1}^{f}W_{ij}SUC_{i,t}\\ +\rho \sum_{j=1}^{f}W_{ij}SAEC_{i,t}+\sum_{n=1}^{k}\sum_{j=1}^{f}\varphi_{n}W_{ij}X_{n,i,t}+\delta_{i}+\mu_{i}+\varepsilon_{i,t}\\ \varepsilon_{i,t}=\lambda \sum_{j=1}^{f}w_{ij}\varepsilon_{j,t}+\tau_{i,t} \end{array}$$ (2)

When $\lambda =0,\rho =0,\varphi_{n}=0$, Eq.(2) is the Spatial Lag Model (SLM); When $\theta =0,\rho =0,\varphi_{n}=0$, Eq.(2) is the Eq.(2) is Spatial Error Model (SEM); and when λ=0, Eq.(2) is the Spatial Durbin Model (SDM). The specific choice of SLM, SEM, or SDM for analysis needs to be determined based on LR test. In addition, $W_{ij}$ is the spatial weight value between province i and province j. And the spatial weight matrix W used in this article is the economic distance matrix.

### 3.2 Indicator selection and data sources

#### 3.2.1 Explained variable.

The explained variable of this article is structural upgrading of the construction industry (SUC).

Construction enterprises can be divided into two types: comprehensive and professional. Comprehensive enterprises mainly refer to construction general contracting enterprises, while professional enterprises refer to enterprises that provide construction production for professional fields, including professional contracting enterprises and labor subcontracting enterprises. The higher the degree of specialization, the more niche markets in the construction market, which can provide various types of construction project engineering and products. At present, the proportion of specialized construction enterprises and comprehensive construction enterprises in China is imbalanced. The number of specialized enterprises is small and not specialized, and the number of comprehensive enterprises is many but not strong, leading to excessive competition in the construction market and hindering the improvement of production efficiency and technological level in the construction industry. Therefore, in order to achieve the SUC, it is necessary to vigorously develop professional construction enterprises and increase their proportion in the entire construction market. This article uses the proportion of professional construction enterprises to the total number of construction enterprises to measure the SUC. The calculation formula is as Eq.(3):


$$SUC_{i,t}=\frac{P_{i,t}}{T_{i,t}}$$ (3)

Where P represents the number of professional contracting units, and T represents the sum of professional contracting and general contracting units.

#### 3.2.2 Core explanatory variable.

The core explanatory variable of this article is structure adjustment of energy consumption (SAEC).

Reducing the proportion of fossil energy consumption while ensuring stable energy supply is an important measure to achieve the “dual carbon” goal. This article takes the change rate of total regional energy consumption (including fossil energy and new energy such as electricity) as a reference object, obtains the actual change rate of fossil energy consumption (including coal, oil, and natural gas) in a certain region, and calculates SAEC. The specific formula is as Eq.(4):


$$SAEC_{i,t}=\frac{TEC_{i,t}}{TEC_{i,t-1}}-\frac{FEC_{i,t}}{FEC_{i,t-1}}$$ (4)

Where $\frac{TEC_{i,t}}{TEC_{i,t-1}}$ represents the change rate of total energy consumption in province i in year t, and $\frac{FEC_{i,t}}{FEC_{i,t-1}}$ represents the change rate of fossil energy consumption in province i in year t. If $SAEC_{i,t}>0$, it represents a decrease in the proportion of fossil energy consumption in year t and an improvement in the energy consumption structure; If $SAEC_{i,t}=0$, it represents that the proportion of fossil energy consumption remains unchanged in year t; If $SAEC_{i,t}<0$, it represents an increase in the proportion of fossil energy consumption in year t and a deterioration in the energy consumption structure.

#### 3.2.3 Control variable.

(1) Foreign direct investment (fdi): Measured by industrial foreign direct investment.
(2) Energy price (ep): Measured by purchasing price indicators for industrial producers of fuel and power.
(3) Investment scale (is): Investment scale is one of the important factors affecting the SUC, especially the scale of fixed assets investment. This paper uses the proportion of fixed assets investment in GDP to measure it.
(4) Human capital (hc): Based on the perspective of education investment, this article regards human capital as the result of education investment and uses education funds to measure it.
(5) Technology R&D investment (tri): Measured by the annual R&D investment of domestic enterprises.

The relevant data in this article are all from the “China Statistical Yearbook”, “China Industrial Economy Statistical Yearbook”, “China Energy Statistical Yearbook”, and “China Construction Industry Statistical Yearbook” from 2008 to 2022. The research object is 30 provinces (autonomous regions, municipalities directly under the central government) in China, but due to data limitations, it does not include the Tibet, Hong Kong, Macau, and Taiwan. The descriptive statistics of the data are shown in Table 1.

**Table 1:** Descriptive statistics.

	Max	Min	Mean	Std. Dev.	Obs.
SUC	0.7700	0.1189	0.3525	0.1405	450
SAEC	0.0762	−0.0865	0.0062	0.0115	450
fdi	10.7204	3.1566	6.5816	1.4705	450
ep	130.2	82.4	101.60	6.6707	450
is	1.4796	0.1998	0.7478	0.2463	450
hc	17.8021	13.0351	15.7913	0.7942	450
tri	17.1836	8.7521	14.1189	1.4416	450

## 4. Empirical results and discussion

### 4.1 Analysis of benchmark regression results

Table 2 shows the regression results of the mixed OLS model, random effects model, fixed effects model, and three spatial econometric models. This article further uses LR test to select the optimal spatial econometric model. The test method significantly rejects the original hypothesis at the 1% level, indicating that the SDM cannot be simplified into an SLM or SEM. Therefore, the analysis is mainly based on the regression results of the SDM.

**Table 2:** Benchmark regression results.

Variables	OLS	RE	FE	SDM	SLM	SEM
	(1)	(2)	(3)	(4)	(5)	(6)
SAEC	0.4747*** (2.76)	0.1532* (1.76)	0.1213*** (2.91)	0.3970*** (2.87)	0.1421** (2.33)	0.5365*** (2.84)
fdi	−0.1749*** (−3.71)	0.0303*** (2.63)	0.0115*** (10.25)	0.0105 (0.93)	0.0059 (0.52)	0.0202 (1.64)
ep	1.0313*** (3.41)	0.5827*** (4.26)	−0.0815*** (−4.18)	−0.3423** (−2.44)	−0.2498* (−1.75)	−0.4490*** (−2.91)
is	0.2549*** (4.56)	−0.0450 (−0.52)	0.0440*** (3.13)	0.0905*** (6.57)	−0.0758*** (−5.05)	0.1374*** (8.55)
hc	0.2474*** (3.53)	0.3725*** (2.95)	0.1275*** (6.79)	0.0388** (2.30)	0.3262*** (8.07)	−0.0245 (−1.19)
tri	0.2633*** (8.91)	0.1976** (2.49)	0.0072 (0.42)	0.0899*** (10.72)	0.0278 (1.44)	0.1148*** (12.44)
W*SAEC				−0.7515*** (−2.71)		
W*fdi				0.0249* (1.90)		
W*ep				−0.3625* (−1.93)		
W*is				0.1808*** (4.91)		
W*hc				0.0936** (2.09)		
W*tri				0.1172*** (6.51)		
*ρ*				−0.3533** (−2.52)	−0.4174*** (−3.70)	−0.5672*** (−5.69)
R_squared	0.7959	0.6573	0.6797	0.6422	0.5754	0.5301
F test			144.54***			
LM test		943.21***				
Hausman test			792.32***			
Fixed effect			YES	YES	YES	YES
Log-L				424.2523	408.8608	372.5613
LR test					92.37***	103.38***
N	450	450	450	450	450	450

Note: Z statistics are in parentheses. ***, **, * represent the significance at 1%, 5%, and 10%, respectively. The same below.

The regression coefficient of SAEC is significantly positive at the 1% level, indicating that the technological effect, price effect, and spatial effect of SAEC on SUC are generally reflected as promoting effects. It means that the optimization of energy consumption structure at present is an effective means to promote the SUC, which is a beneficial supplement to relevant research. On the premise of ensuring stable economic growth, forcing the SUC through the control of total fossil energy consumption and intensity requires a relatively long-term process. However, with the urgent need for energy transformation and the deepening of new energy development strategies, government departments use energy quotas, subsidies and other means to restrict fossil energy and encourage the use of new energy, resulting in investment incentives, technological innovation, and new energy substitution, which will be internalized in the process of optimizing the energy consumption structure, ultimately effectively promoting the SUC.

Quantitatively, a 1% increase in SAEC is associated with an approximate 0.397% increase in the proportion of professional construction enterprises. In practical terms, this means that for a province with 10,000 construction enterprises, SAEC growth of 1% would lead to an additional 39–40 professional contracting units, reflecting a shift toward more specialized, technology-intensive segments (e.g., green building engineering, smart construction) and a decline in high-energy-consuming general contracting units. This structural shift aligns with the goal of reducing the construction industry’s carbon intensity while improving productivity.

Beyond statistical significance, the economic impact of SAEC is substantial. Given that professional enterprises have 15–20% higher labor productivity than general contracting units, this translates to an estimated 0.06–0.08% increase in the construction industry’s total factor productivity. For a province with annual construction output of 1 trillion yuan, this would imply additional output value of 6–8 billion yuan, highlighting the tangible economic benefits of energy structure adjustment.

From the perspective of spatial effect, firstly, the regression coefficient of W*SAEC is significantly negative at the 1% level, indicating that the SAEC has a negative spatial spillover effect on the SUC. That is to say, due to the differences in the SAEC between regions, the high-energy consuming construction industry tends to shift towards regions with relatively loose energy consumption regulations, promoting the differentiation of the construction industry structure between regions. The spatial effect in theoretical analysis are empirically supported. Secondly, the spatial lag coefficient *ρ* of the SUC is significantly negative at the 5% level, indicating that the construction industry structure between regions also has a spatial negative spillover effect. On the one hand, as mentioned earlier, SAEC promotes the spatial transfer between high-energy consuming construction industry and high-tech construction industry, which is an important source of negative spatial spillover effects. On the other hand, when the government intervenes in industrial layout for growth goals or fiscal revenue, different regions will promote the deepening of regional division of labor through differentiated characteristic industrial policies. Therefore, the construction industry between regions also enjoys the positive spatial spillover effect generated by coordinated development. From the final result, the negative spatial spillover effect of the current construction industry structure dominates.

### 4.2 Analysis of endogeneity

The SUC has led to a decrease in fossil energy consumption, which may have a negative impact on the SAEC. This article intends to use the instrumental variable method to solve the endogeneity problems that may exist in the model. Specifically, the SAEC with a lag of one period (L.SAEC) and air flow coefficient (afc) are selected as instrumental variables. The reason for selecting the afc is that as a natural condition, the afc does not directly affect the structure of the construction industry and meets the condition of exogenous instrumental variables [30]. At the same time, the larger the afc, the faster the diffusion rate of atmospheric pollutants. The externalities of pollution may lead to a decrease in the motivation of local governments to control pollution emissions, thereby weakening their energy consumption structure optimization efforts and meeting the conditions of instrumental variable correlation. Table 3 shows the regression results of the two-stage least squares method, from which it can be seen that the F-values of the two instrumental variables in the first stage are significantly greater than 10, which conforms to the rule of thumb that is not a “weak instrumental variable”. From the regression results of the second stage, it can be seen that the SAEC under both instrumental variables has a significant positive effect on the SUC, which is consistent with the regression results mentioned above. Therefore, after considering the potential endogeneity issues in the model, the current SAEC has indeed played a role in promoting the SUC. The core conclusion of this article is relatively stable.

**Table 3:** Endogeneity results.

Variables	IV: L.SAEC		IV:afc	
	First stage	Second stage	First stage	Second stage
L.SAEC	0.9329*** (39.12)			
afc			0.9518*** (28.41)	
SAEC		0.1472** (2.36)		0.0044*** (4.11)
Control variable	YES	YES	YES	YES
Fixed effect	YES	YES	YES	YES
F value	146.53		143.50	
R_squared	0.6835		0.6797	
N	420		450	

### 4.3 Robustness checks

To verify the robustness of the benchmark regression results, the system GMM (SYS-GMM) and differential GMM (DIFF-GMM) methods were used for testing. Considering the possible dynamic endogeneity between the SUC and SAEC, that is, the current SUC may be affected by its lagged value, the system GMM effectively alleviates the weak instrumental variable problem and improves parameter estimation efficiency by combining differential GMM and horizontal GMM. The results in Table 4 show that the coefficient of the SUC lag term (L.SUC) is significantly positive at the 1% level, indicating that the structural upgrading of the construction industry has strong path dependence; At the same time, the SAEC coefficient was significantly positive at the 1% level in both the DIFF-GMM and SYS-GMM models, and both AR and Sargan tests validated the effectiveness of instrumental variables, indicating that after controlling for dynamic endogeneity, the promoting effect of SAEC on SUC remained robust.

**Table 4:** Robustness checks.

Variables	DIFF-GMM	SYS-GMM	SUC
L.SUC	0.9861*** (71.31)	0.9108*** (47.72)	
SAEC	0.2745*** (3.00)	0.0046*** (5.92)	0.2013*** (4.62)
Control variable	YES	YES	YES
Fixed effect	YES	YES	YES
AR(1)	0.0107	0.0205	
AR(2)	0.8209	0.8081	
Sargan检验	0.5887	0.6017	

In addition, robustness tests were conducted by removing extreme value samples (truncated by 1% and 99%), and values below the 1% percentile and above the 99% percentile were replaced with corresponding percentile values to eliminate the interference of extreme values on the regression results. The results in the column (3) of Table 4 show that the processed SAEC coefficient is still significantly positive at the 1% level, consistent with the baseline regression conclusion, further confirming that the promotion effect of energy consumption structure adjustment on the upgrading of the construction industry structure is stable and not affected by extreme values.

### 4.4 Function channel inspection

In addition to exploring the spatial effects of SAEC on SUC based on spatial econometric models, this section examines the channels of SAEC on SUC from the perspectives of technical effect and price effect, as described in the theoretical mechanism. Among them, technical effect is measured from two perspectives: foreign direct investment (fdi) and technology R&D investment (tri), respectively representing technology spillovers and technology R&D channels. Price effect is measured by energy price (ep). Referring to the approach of Zou et al. [31], in order to alleviate the endogeneity problem of bidirectional causality between SAEC and technology spillover, technology R&D, and energy price, the SAEC with a lag of one period (L.SAEC) was used to substitute the model for regression. The results are shown in Table 5.

**Table 5:** Results of function channel inspection.

Variables	Technology spillover		Technology R&D		Energy price	
	(1)fdi	(2)SUC	(3)tri	(4)SUC	(5)ep	(6)SUC
L.SAEC	0.7425*** (2.64)		−0.8222*** (−3.26)		−0.9429 (−0.12)	
fdi		0.0216** (2.19)				
tri				0.1603*** (2.68)		
ep						2.2679 (1.13)
Control variable	YES	YES	YES	YES	YES	YES
Fixed effect	YES	YES	YES	YES	YES	YES
R_squared	0.2711	0.8995	0.1956	0.8995	0.3465	0.8995
Log-L	618.9224	92.1662	642.9049	92.1662	967.6920	92.1662
N	420	450	420	450	420	450

Note: The regression model is SDM, with z values in parentheses (the same below).

In column (1) and column (3), the regression coefficient of SAEC on technology spillover is significantly positive, while the regression coefficient on technology R&D is significantly negative. The above results indicate that under energy consumption constraints, enterprises will be forced to directly introduce advanced foreign technologies or learn from mature development models to save development costs and trial and error costs. However, it may hinder the crowding out effect of cost pressure on the company’s own tri. The negative impact of SAEC on domestic tri can be explained by two mechanisms. Firstly, short-term cost pressure. Adjusting energy consumption structure (e.g., reducing fossil energy use and adopting new energy) increases immediate operational costs for construction enterprises, such as investments in new energy equipment, retrofitting production lines, and compliance with stricter energy standards. These costs squeeze the budget allocated to long-term R&D, which has uncertain and delayed returns. Secondly, substitution effect of external technology. Under energy constraints, enterprises tend to prioritize importing mature foreign technologies (reflected in the positive effect on technology spillover, fdi) over developing independent innovations. This is because external technologies can quickly meet energy-saving requirements with lower trial-and-error costs, reducing incentives for local R&D. Thus, SAEC crowds out domestic R&D in the short term due to cost competition and the convenience of substituting with external technologies, though this effect may weaken as enterprises adapt to new energy structures and shift focus to innovation.

Furthermore, from the regression results in column (2) and column (4), it can be seen that the regression coefficients for technology spillover and technology R&D are significantly positive, indicating that both are the main driving forces for promoting the SUC. Overall, the SAEC has enhanced the degree of technology spillover to promote the SUC, while weakening the promoting effect of technology R&D on the SUC. In addition, from column (5) and column (6), it can be seen that the impact of SAEC on energy price is not significant, which may be related to government regulation of energy price. Energy price reform in China has long been characterized by delayed and reactive adjustments rather than proactive market-oriented reforms. For instance, fossil energy prices (e.g., coal and natural gas) are often regulated by the government to stabilize the economy, leading to a lag in their response to changes in supply and demand or environmental costs. This slowness and inertia in price adjustment weaken the role of energy prices as a market signal, resulting in an insignificant price effect channel.

### 4.5 Further discussion

#### 4.5.1 Heterogeneity test of changes in the proportion of various energy consumption.

Based on the measurement method of Eq.(4), the consumption structure adjustment indices of coal (S1), petroleum (S2), natural gas (S3), and new energy (S4) were calculated to analyze their heterogeneity impact on the SUC. The regression results of the SDM model are shown in Table 6. Firstly, as shown in column (1) and column (2), the regression coefficients of S1 and S2 are significantly positive, indicating that reducing their proportion in total energy consumption can promote the SUC, which is consistent with the meaning expressed by the benchmark regression results. As shown in column (3), the finding that reducing natural gas consumption promotes SUC, though seemingly counterintuitive given natural gas is widely regarded as a cleaner fossil fuel, is rooted in three contextual realities of China’s construction industry during the study period. First, natural gas consumption in China’s construction sector is disproportionately concentrated in energy-intensive subfields, such as cement kilns, steel smelting for building materials, and traditional heating systems, all dominated by low-value-added general contracting enterprises. These enterprises rely on relatively cheap natural gas (partly due to government subsidies) to maintain large-scale production, and their expansion directly crowds out resources for professional high-tech segments. Reducing natural gas consumption thus curbs the scale of such low-efficiency activities, creating space for SUC. Second, China’s energy endowment, which is characterized by “rich coal, poor oil, little gas”, means natural gas supply is heavily dependent on imports. Sustained growth in natural gas consumption for high-energy construction activities exacerbates energy security risks, while limiting it forces enterprises to either upgrade technologies or exit, accelerating structural replacement. Third, during 2008–2022, natural gas infrastructure in China’s construction sector remained underdeveloped, with limited application in high-tech green technologies. Most consumption served traditional energy-intensive processes, so reducing it targeted backward capacity without hindering innovative segments. In addition, the regression coefficients of the spatial lag terms W*S1 and W*S3 are significantly negative, once again confirming the spatial negative spillover effect of SAEC in the theoretical mechanism analysis above. Secondly, as shown in column (4), the regression coefficient of S4 is significantly positive, indicating that the increase in the proportion of new energy consumption promotes the SUC. The reason behind it is that increasing the proportion of new energy consumption means increasing the demand for new energy, thereby promoting the development of the new energy industry. As a high-tech industry, it can significantly promote the local SUC.

**Table 6:** Heterogeneity test results of changes in the proportion of various energy consumption.

Variables	Coal	Petroleum	Natural gas	New energy
	(1)	(2)	(3)	(4)
S1	0.6844*** (3.31)			
S2		0.0012* (1.69)		
S3			1.3961*** (2.61)	
S4				0.7447*** (2.57)
W*Si	−0.5745*** (−2.70)	−0.2795 (−0.25)	−0.0511** (−2.02)	−0.8217 (1.00)
*ρ*	−0.0825*** (13.96)	−0.1380** (−1.96)	−0.0276** (−2.09)	−0.0804** (−2.01)
Control variable	YES	YES	YES	YES
Fixed effect	YES	YES	YES	YES
R_squared	0.5839	0.2223	0.3007	0.5244
Log-L	729.4181	111.7331	357.7765	596.0534
N	450	450	450	450

#### 4.5.2 Spatiotemporal heterogeneity test.

To further explore the objective laws of SAEC on SUC, this article divides 30 provinces into the eastern region (Beijing, Hainan, Fujian, Shandong, Guangdong, Hebei, Jiangsu, Liaoning, Tianjin, Shanghai, Zhejiang) and the central and western regions (the remaining provinces). From the perspective of regional heterogeneity and temporal heterogeneity, analysis is conducted. Table 7 shows the regression results based on the SDM model.

**Table 7:** Results of spatiotemporal heterogeneity test.

Variables	Spatial heterogeneity		Temporal heterogeneity	
	(1)Eastern	(2)Central and western	(3)2008-2014	(4)2015-2022
SAEC	0.1272 (0.89)	1.1798*** (3.36)	1.6433* (1.71)	0.2191*** (3.17)
W*SAEC	−0.6625** (−2.16)	−0.5199*** (−4.40)	−1.0616 (−1.63)	−0.7136*** (−3.30)
*ρ*	−0.1964*** (8.71)	−0.2377** (−2.22)	−0.0119*** (−4.25)	−0.0480** (−2.73)
Control variable	YES	YES	YES	YES
Fixed effect	YES	YES	YES	YES
R_squared	0.8046	0.4371	0.7864	0.6837
Log-L	353.3666	316.6962	339.4969	240.4808
N	165	285	210	240

(1) Regional heterogeneity. Table 7 shows distinct spatial patterns in the impact of SAEC on SUC. Specifically, the central and western regions exhibit a significantly stronger promoting effect (SAEC coefficient = 1.1798, p < 0.01) compared to the eastern region (SAEC coefficient = 0.1272, insignificant). This indicates that energy consumption structure adjustment drives more pronounced upgrading of the construction industry in central and western provinces—consistent with their higher dependence on fossil energy, making them more sensitive to structural changes. In terms of spatial spillovers, the eastern region shows a larger negative spillover effect (W*SAEC coefficient = −0.6625, p < 0.05) than the central and western regions (W*SAEC coefficient = −0.5199, p < 0.01). This aligns with the uneven distribution of energy regulations: eastern provinces with stricter fossil energy controls push high-energy-consuming construction enterprises to relocate to neighboring areas with looser policies, exacerbating regional disparities in industry structure.
(2) Temporal heterogeneity. The regression coefficient of SAEC was significantly positive in both periods, but the promotion effect of SAEC on SUC was stronger in the previous period. In the previous period, the proportion of high-energy consuming construction industry was relatively high, and the SAEC helped to induce the transfer of factors such as technology, labor, and capital to high-tech industries. Over time, the bottleneck constraints of SUC have increased, relying more and more on independent innovation capabilities. Therefore, the effect of SAEC shows a trend of diminishing marginal effects. From the perspective of spatial effects, W*SAEC was only significantly negative in the later period. On the one hand, with the passage of time, the new energy power supply in the western region urgently needs to be absorbed by the high-energy consuming construction industry, while the constraints on fossil fuels such as coal and oil in the eastern region are becoming increasingly tight, leading to an increasing trend of the transfer of the high-energy consuming construction industry to the western new energy province. On the other hand, with the improvement of infrastructure such as transportation and information, the compression of spatiotemporal distance has reduced the attractiveness of developed coastal areas. Therefore, driven by the SAEC, the high-energy consuming construction industry is more inclined to transfer to surrounding underdeveloped areas.

#### 4.5.3 Impact of external environment.

On the basis of Eq.(1) and Eq.(2), the interaction terms of SAEC*ep and SAEC*hc are gradually added to explore the impact of external environment. To make the regression coefficients of the variables meaningful at the same time, the data was centralized before taking the interaction terms. The regression results are shown in Table 8. It can be seen that the regression results of SAEC are still significantly positive in all models, and the regression coefficients of ep and hc are also consistent with the previous results. From the regression coefficient of the interaction term, the coefficient of SAEC*ep is significantly positive in the FE and SDM, indicating that the impact of SAEC on SUC is to some extent positively regulated by energy price. It indicates that, with a fixed degree of SAEC, appropriately increasing energy price is conducive to further unleashing the promoting effect of SAEC on SUC. Similarly, the coefficient of SAEC*hc is significantly positive, indicating a positive moderating effect on human capital level. It means that with the improvement of human capital level, the promoting effect of SAEC on SUC has been further strengthened, reflecting the important role of technological progress and intellectual support brought by human capital in promoting the SUC [32,33].

**Table 8:** Test results of external environmental impacts such as energy price and human capital.

Variables	ep		hc	
	(1)FE	(2)SDM	(3)FE	(4)SDM
SAEC	0.1689** (2.48)	0.0870*** (2.63)	0.9319** (2.08)	0.6737** (2.13)
ep	−0.1702 (−1.40)	−0.5562 (−0.60)		
SAEC*ep	0.9284*** (2.91)	0.9005** (2.40)		
hc			0.5352*** (11.05)	1.0604*** (6.51)
SAEC*hc			0.8507* (1.89)	0.3656*** (3.39)
Constant	6.3055*** (11.27)		−1.2640* (1.71)	
Control variable	YES	YES	YES	YES
Fixed effect	YES	YES	YES	YES
R_squared	0.7792	0.8095	0.7968	0.8671
Log-L		103.1107		148.5805
N	450	450	450	450

## 5. Conclusion and policy implications

This article explains the theoretical mechanism of SAEC on SUC from three aspects (technical effect, price effect, and spatial effect), and then conducts empirical testing. The research results indicate that:

At the national level, SAEC has a significant promoting effect on SUC, and after endogeneity analysis using instrumental variable method, the promoting effect remains robust. In terms of spatial effects, SAEC has a significant negative spillover effect on SUC, and SAEC also has an impact on SUC through technology spillover and technology R&D, but the mechanism of price effect is not obvious. Further research has found that reducing the proportion of coal, oil, and natural gas consumption can promote SUC, with natural gas having the strongest effect, while increasing the proportion of new energy consumption has a weak promoting effect on SUC. At the regional level, SAEC has a stronger promoting effect on SUC in the central and western regions, but its spatial negative spillover effect is more pronounced in the eastern region. At the time level, it shows a decreasing trend in marginal effects. From 2008 to 2014, SAEC had a stronger promoting effect on SUC, while its spatial negative spillover effect was more pronounced in 2015–2022. In addition, optimizing the external environment such as human capital levels and energy prices will further enhance the promoting effect of SAEC.

Based on the research conclusions of this article, the following policy implications can be obtained:

(1) Promote the continuous optimization of energy consumption structure. Firstly, continuously develop and utilize new energy sources, such as increasing the proportion of solar power generation, promoting the use of biological intelligence, and supporting the development of new energy vehicles. Secondly, curb high carbon energy consumption and improve the competitiveness of cleaner energy. Finally, the focus is on strengthening the management of natural gas consumption. China’s energy endowment is characterized by “rich coal, poor oil, and little gas”, and its natural gas reserves are insufficient. The reduction in the proportion of natural gas consumption can help promote the SUC.
(2) Construct a coordinated development pattern for the regional construction industry. Pay attention to the interactive mechanism of the construction industry at the spatial level, especially in the eastern region. Promote the formulation of industrial coordinated development plans between regions with close geographical locations and similar resource endowments, so as to leverage the radiation and drive of high-level areas of the construction industry to low-level areas. For the central and western regions, the structural level of their construction industry is relatively low. It should leverage their comparative advantages such as low production factor costs, actively undertake industrial transfer and introduce advanced technologies, promoting SUC.
(3) Promote the coordination of energy policies with the construction industry structure. The dependence on fossil fuels in the eastern region is relatively weak, and in the future, efforts should continue to increase the optimization of energy consumption structure in the eastern region to improve the marginal effect of SAEC on SUC. For the western region, the sufficient supply of new energy leads to significant consumption pressure. Therefore, it is necessary to moderately promote the transfer of energy intensive construction industry in the eastern region to the central and western regions, achieve spatial coupling of new energy supply and demand. At the same time, it should actively guide and encourage the agglomeration and development of high-tech construction industry in the upstream and downstream of new energy, and use the demand effect of new energy to drive the SUC.
(4) Integration of international experience and localized adaptation. Formulate regional mandatory standards for the utilization rate of new energy in the construction industry (e.g., 30% in eastern regions by 2025 and 20% in central and western regions by 2030), and provide carbon emission rights trading quota rewards to enterprises that overfulfill the targets. Incorporate energy structure adjustment into the “assessment of high-quality development of the construction industry” for local governments, while adding “technical assistance” clauses, such as the transfer of new energy building technologies from eastern regions to central and western regions. Introduce Germany’s energy-saving technologies for prefabricated buildings and Denmark’s experience in wind energy application in buildings, and at the same time export China’s low-cost solutions for building-integrated photovoltaics to form two-way technological spillover. Connect with the “Building Decarbonization Alliance” under the Paris Agreement, and incorporate China’s experience in regional heterogeneity into the international policy reference framework. In addition, China needs to balance multiple new energy sources such as solar energy, wind energy, and biomass energy to avoid structural vulnerability caused by over-reliance on a single energy source.

Under China’s “dual carbon” goals, a dual control system has been implemented for both the total amount and intensity of energy consumption. Local governments exercise mandatory quota management over fossil energy consumption. This top-down policy-driven model differs from the market-oriented energy regulatory systems in Europe and the United States, which may have strengthened the forcing effect of energy consumption structure adjustment on industrial upgrading. China’s construction industry features a distinct “government-led” development model, where large-scale projects rely on government investment, and there are strong administrative interventions in areas such as industry access and qualification management. In addition, the long-term low proportion of professional enterprises in China’s construction industry leads to a significant difference in market structure compared with developed countries (e.g., Singapore, the United Kingdom). This may make the role of energy structure adjustment in promoting professional upgrading more prominent. The research conclusions of this paper are of high reference value for transition economies (e.g., India, Brazil), as they face similar pressures of fossil energy dependence and industrial upgrading. However, for developed countries with a high degree of energy marketization and mature construction industries, policy implications need to be adjusted in combination with their market mechanisms.

However, this article also have some limitation. Firstly, the measurement of human capital in this study relies on education investment, which may not fully capture the actual skill levels of the construction workforce. Factors such as on-the-job training, technical certifications, and practical experience—key indicators of workforce quality in the construction industry—are not included, potentially weakening the precision of the human capital channel analysis. Future research could integrate micro-level data on worker skills (e.g., vocational qualification statistics) to improve measurement accuracy. Secondly, although the afc satisfies the exogeneity and correlation conditions for an instrumental variable, it has a limitations: the afc is closely related to regional topography and climate (e.g., wind speed, terrain relief), which may vary significantly across countries. Its validity as an instrument is primarily applicable to China’s provincial panel data, limiting the generalizability of findings to other regions with different geographical characteristics.

## Supporting information

S1 Data Supporting information. (XLSX)

## References

[1] Zhang H, Li L, Chen T, Li V. Where will China’s real estate market go under the economy’s new normal? Cities. 2016;55:42–8. doi: 10.1016/j.cities.2016.03.014

[2] Liu Y, Liu M, Wang G, Zhao L, An P. Effect of environmental regulation on high-quality economic development in china-an empirical analysis based on dynamic spatial durbin model. Environ Sci Pollut Res Int. 2021;28(39):54661–78. doi: 10.1007/s11356-021-13780-2 34018107

[3] Zuo J, Zhao Z-Y. Green building research–current status and future agenda: a review. Renew Sustain Energy Rev. 2014;30:271–81. doi: 10.1016/j.rser.2013.10.021

[4] Jaafar MH, Arifin K, Aiyub K, Razman MR, Ishak MIS, Samsurijan MS. Occupational safety and health management in the construction industry: a review. Int J Occup Saf Ergon. 2018;24(4):493–506. doi: 10.1080/10803548.2017.1366129 28849991

[5] Hanif I. Impact of fossil fuels energy consumption, energy policies, and urban sprawl on carbon emissions in East Asia and the Pacific: a panel investigation. Energy Strategy Rev. 2018;21:16–24. doi: 10.1016/j.esr.2018.04.006

[6] Yang J, Cai W, Ma M, Li L, Liu C, Ma X, et al. Driving forces of China’s CO2 emissions from energy consumption based on Kaya-LMDI methods. Sci Total Environ. 2020;711:134569. doi: 10.1016/j.scitotenv.2019.134569 32000310

[7] Mi Z-F, Pan S-Y, Yu H, Wei Y-M. Potential impacts of industrial structure on energy consumption and CO2 emission: a case study of Beijing. J Clean Prod. 2015;103:455–62. doi: 10.1016/j.jclepro.2014.06.011

[8] Zhu B, Zhang T. The impact of cross-region industrial structure optimization on economy, carbon emissions and energy consumption: a case of the Yangtze River Delta. Sci Total Environ. 2021;778:146089. doi: 10.1016/j.scitotenv.2021.146089 34030353

[9] Hong Y, Can P, Xiaona Y, Ruixue L. Does change of industrial structure affect energy consumption structure: a study based on the perspective of energy grade calculation. Energy Explor Exploit. 2018;37(1):579–92. doi: 10.1177/0144598718784032

[10] Neagu O, Haiduc C, Anghelina A. Does renewable energy matter for economic growth in central and Eastern European countries? Empirical evidence from heterogeneous panel cointegration analysis. Studia Univ Vasile Goldis Arad – Econ Ser. 2021;31(1):34–59. doi: 10.2478/sues-2021-0003

[11] Ren S, Hao Y, Xu L, Wu H, Ba N. Digitalization and energy: how does internet development affect China’s energy consumption? Energy Econ. 2021;98:105220. doi: 10.1016/j.eneco.2021.105220

[12] Zeng S, Su B, Zhang M, Gao Y, Liu J, Luo S, et al. Analysis and forecast of China’s energy consumption structure. Energy Policy. 2021;159:112630. doi: 10.1016/j.enpol.2021.112630

[13] Ofori G. International contractors and structural changes in host country construction industries: case of Singapore. Eng Constr Archit Manag. 1996;3(4):271–88. doi: 10.1108/eb021035

[14] Tian Y, Xiong S, Ma X. Analysis of the potential impacts on China’s industrial structure in energy consumption. Sustainability. 2017;9(12):2284. doi: 10.3390/su9122284

[15] Woo TH. Climate change modelling for nuclear industry in the aspect of energy consumption using system dynamics method. IJGW. 2018;16(2):136. doi: 10.1504/ijgw.2018.094553

[16] Sajadifar S, Mohamadbagheri A. Environmental performance of OPEC countries: addressing the role of gross domestic product, energy consumption, trade openness and industrial structure. IJGE. 2018;12(3/4):258. doi: 10.1504/ijge.2018.097870

[17] Zhu B, Shan H. Impacts of industrial structures reconstructing on carbon emission and energy consumption: a case of Beijing. J Clean Prod. 2020;245:118916. doi: 10.1016/j.jclepro.2019.118916

[18] Taherahmadi J, Noorollahi Y, Panahi M. Toward comprehensive zero energy building definitions: a literature review and recommendations. Int J Sustain Energy. 2020;40(2):120–48. doi: 10.1080/14786451.2020.1796664

[19] Wu X, Zhu R, Wang T, Chen Y. Market or Mandate? Exploring synergistic low‐carbon regulations and green‐biased technological progress. Manage Decis Econ. 2025. doi: 10.1002/mde.70004

[20] Prasad CS, Saxena A, Dutta D. Building bridges in policy implementation during a pandemic: insights from an e-survey on Indian Producer Organisations. Dev Pract. 2023;33(7):841–51. doi: 10.1080/09614524.2023.2237210

[21] Yan J, Zhao T, Lin T, Li Y. Investigating multi-regional cross-industrial linkage based on sustainability assessment and sensitivity analysis: a case of construction industry in China. J Clean Prod. 2017;142:2911–24. doi: 10.1016/j.jclepro.2016.10.179

[22] Saif S, Wang Y, Iqbal S, Amin N, Mushtaque I. Paradox of achieving sustainable development goals by 2030 in gulf cooperation council: differential effects of technological innovation, renewable energy and bio-capacity on carbon emission. Clean Techn Environ Policy. 2025;27(7):3021–40. doi: 10.1007/s10098-024-03118-0

[23] Antimiani A, Costantini V, Paglialunga E. Fossil fuels subsidy removal and the EU carbon neutrality policy. Energy Econ 2023;119:106524. doi: 10.1016/j.eneco.2023.106524

[24] Pradhan BK, Ghosh J. A computable general equilibrium (CGE) assessment of technological progress and carbon pricing in India’s green energy transition via furthering its renewable capacity. Energy Econ. 2022;106:105788. doi: 10.1016/j.eneco.2021.105788

[25] Franco MAJQ, Pawar P, Wu X. Green building policies in cities: a comparative assessment and analysis. Energy Build 2021;231:110561. doi: 10.1016/j.enbuild.2020.110561

[26] Lin B, Zhou Y. How does vertical fiscal imbalance affect the upgrading of industrial structure? Empirical evidence from China. TFSC. 2021;170:120886. doi: 10.1016/j.techfore.2021.120886

[27] Zhou X, Feng C. The impact of environmental regulation on fossil energy consumption in China: direct and indirect effects. J Clean Prod. 2017;142:3174–83. doi: 10.1016/j.jclepro.2016.10.152

[28] He Y, Lin B. The impact of natural gas price control in China: a computable general equilibrium approach. Energy Policy. 2017;107:524–31. doi: 10.1016/j.enpol.2017.05.015

[29] Yong JY, Yusliza M-Y, Jabbour CJC, Ahmad NH. Exploratory cases on the interplay between green human resource management and advanced green manufacturing in light of the Ability-Motivation-Opportunity theory. JMD. 2019;39(1):31–49. doi: 10.1108/jmd-12-2018-0355

[30] Wu H, Hao Y, Ren S, Yang X, Xie G. Does internet development improve green total factor energy efficiency? Evidence from China. Energy Policy. 2021;153:112247. doi: 10.1016/j.enpol.2021.112247

[31] Zou X, Yang X, Jiang JL. The impact of energy consumption structure adjustment on the upgrading of manufacturing industry structure: an empirical analysis based on manufacturing industry segmentation. Soft Sci. 2023:1–13.

[32] Huang C, Zhang X, Liu K. Effects of human capital structural evolution on carbon emissions intensity in China: a dual perspective of spatial heterogeneity and nonlinear linkages. Renew Sustain Energy Rev. 2021;135:110258. doi: 10.1016/j.rser.2020.110258

[33] Wang X, Wang Q. Research on the impact of green finance on the upgrading of China’s regional industrial structure from the perspective of sustainable development. Resour Policy. 2021;74:102436. doi: 10.1016/j.resourpol.2021.102436

